# A simple new technique for the removal of fractured femoral stems: a case report

**DOI:** 10.1186/1752-1947-8-151

**Published:** 2014-05-15

**Authors:** Goran Bicanic, Kresimir Crnogaca, Domagoj Delimar

**Affiliations:** 1 Department of Orthopaedic Surgery, University of Zagreb School of Medicine, Clinical Hospital Centre Zagreb, Salata 6-7, 10000, Zagreb, Croatia; 2 Department of Orthopaedic Surgery, Clinical Hospital Centre Zagreb, Salata 7, 10000, Zagreb, Croatia

**Keywords:** Cementless femoral stem, Stem breakage, Stem fracture, Revision surgery, Extraction

## Abstract

**Introduction:**

The removal of broken femoral stems has become a major issue in revision surgery, and is a technically difficult and time-consuming procedure.

**Case presentation:**

We present a case of a fracture of a cementless long femoral stem in a 65-year-old, white Caucasian man. The distal part was removed with a special longitudinal osteotomy through the anterior cortex extending distally for 10cm. It was then followed by a transversal osteotomy 2cm below the tip of the femoral stump to allow enough space for two locking pliers. Simultaneously using a lamina spreader on the distal part, the broken stem was extracted while hammering on two locking pliers.

**Conclusions:**

We developed a simple and easy technique for the removal of a broken femoral stem that can be applied to all kinds of femoral stems and intramedullary nails regardless of their cross section. We used ordinary surgical instruments and spared the remaining bone stock.

## Introduction

One of the modes of failure of a femoral prosthesis is stem breakage. The removal of broken femoral stems has become a major issue in revision surgery. The extraction of a well-fixed femoral component can be extremely demanding and time-consuming and can result in damage to the remaining bone stock. Various techniques have been developed for the removal of well-fixed cementless femoral stems [1,2]. Technical issues present in such operations are: 1) providing a firm grip of the stem, and 2) the manner of extraction of the stem with conservation of bone stock.

We present a case of fracture of a cementless long femoral stem, which is mainly used in tumor and revision surgery, and a useful technique to remove it from the intramedullary space of the femur.

## Case presentation

The patient, a 65-year-old, white, Caucasian man with a height of 172cm, weight of 75kg and body mass index (BMI) of 25.35kg/m2, was admitted to our clinic for the first time in June 1999 when a biopsy of the left hip was done. It was diagnosed as a myxoid fibrosarcoma (low grade G2). Following the biopsy, our patient underwent a resection of a tumor and the femur 12cm in length, and a reconstruction with MP^™^ Reconstruction Hip Stem (Waldemar Link GmbH & Co KG, Hamburg, Germany) and HI^™^ Acetabulum (Intraplant, Cham, Switzerland). The femoral component was a cementless stem made of titanium alloy with distal anchoring and had longitudinal fluting to provide rotational stability. It was modular with a porous-coated surface to promote bone ingrowth. The postoperative course was without complications. Our patient underwent regular ambulatory controls with clinical and radiology examinations annually. He exerted high levels of physical activity. In October 2012, our patient felt a sharp pain in his left hip while walking in the street, which prevented him from continuing to walk. After a clinical examination and a review of his radiographs, a diagnosis of fracture of the femoral stem was made. Our patient’s left leg was immobilized and put into traction. After standard preoperative preparations, our patient had his first operation in the lateral decubitus position using spinal anesthesia. The fracture was reached with the direct lateral approach extended distally. Below the fascia, proximally around his hip, hard white-yellow tumor tissue was found that extended in the inguinum and dorsal, close to the nervus ischiadicus. It was suspected to be a recurrence of a primary tumor. The tumor tissue, 11.5cm in length and 7cm in width, was excised and sent for pathohistological analysis. Microbiological samples were taken as well. The definitive reconstruction was postponed. Our patient underwent a multislice spiral computed tomography (MSCT) scan of his pelvis and lower left extremity, and a bone scan with Tc-99m methylene diphosphonate (Tc-99m-MDP). Both demonstrated a local recurrence of the primary tumor without propagation in his pelvis. Microbiological samples were sterile and C-reactive protein (CRP) and erythrocyte sedimentation rate (ESR) were within the range of normal values. A biopsy suggested a definitive diagnosis of low-grade fibromyxoid sarcoma and our patient was prepared for a definitive operative procedure.

### Operative technique

**Figure 1:**
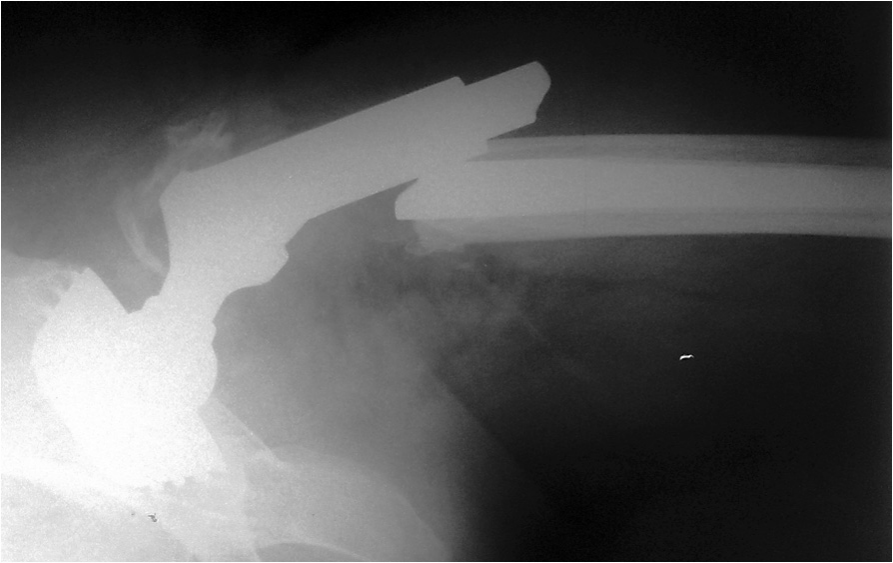
Plain radiograph of the fractured prosthesis.

**Figure 2:**
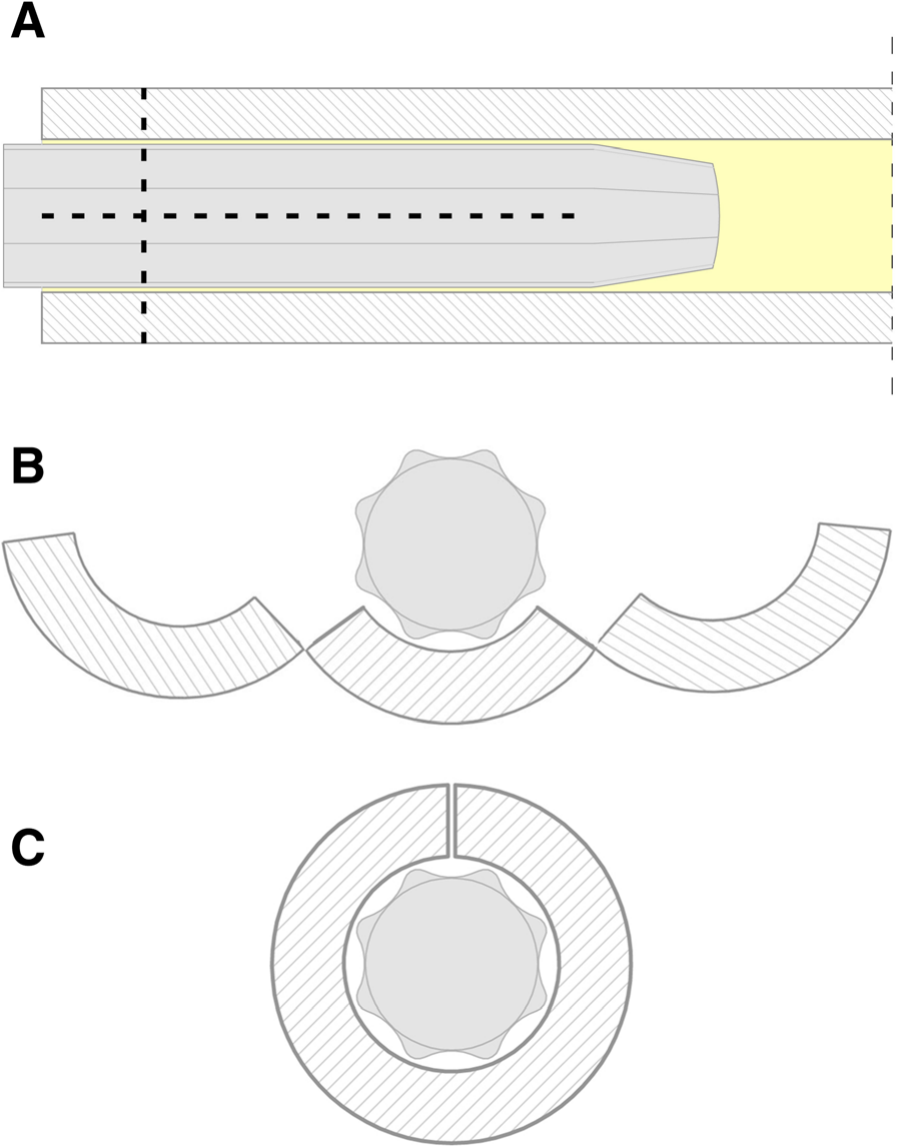
**Osteotomy lines. (A)** Osteotomy lines in the coronal plane. Longitudinal osteotomy extending distally until the narrowing of the prosthesis. **(B)** Osteotomy lines in the transversal plane at the proximal tip of the broken stem. Bone flakes opened like a book. **(C)** Osteotomy lines in the transversal plane at the distal part of the broken femoral stem.

**Figure 3:**
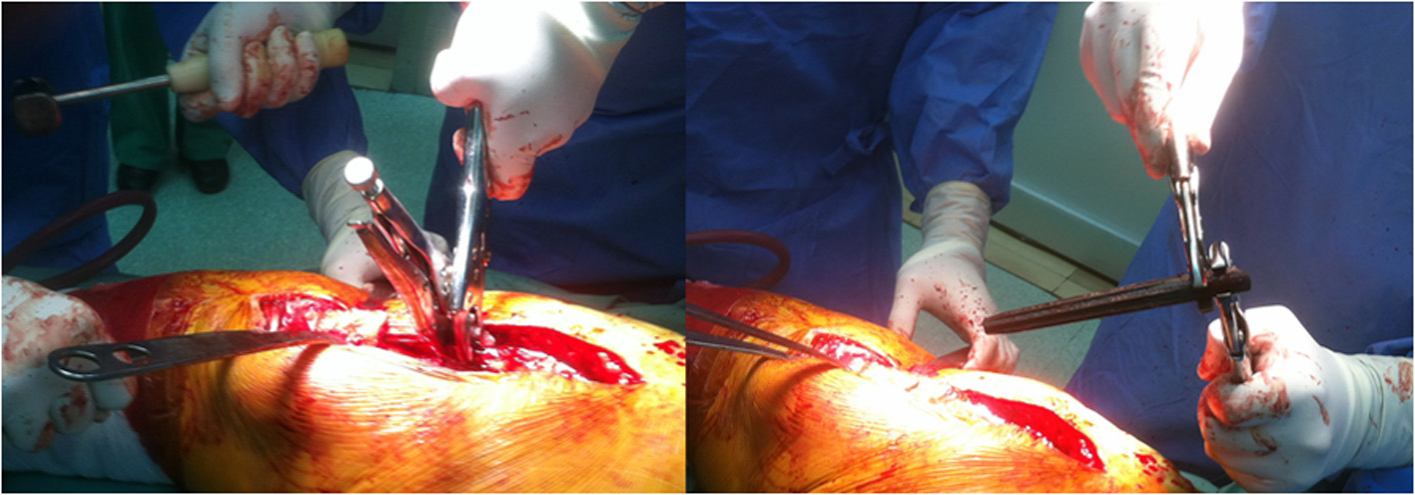
Intraoperative photographs.

**Figure 4:**
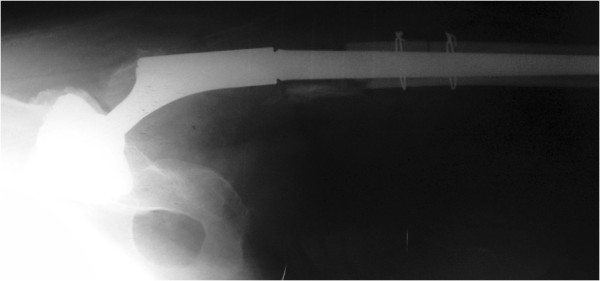
**Postoperative plain radiograph.** Note the excellent adaptation of the osteotomy lines.

Our patient was given spinal anesthesia, in the lateral decubitus position. We used the direct lateral approach that was extended distally. The fracture was 7cm distally from the tip of the greater trochanter, just at the junction of the proximal and distal modular parts and at the level of the remaining bone after the primary resection (Figure 1). The proximal part of the femoral stem was extracted easily and without complications. We continued with the disengagement of the proximal portion of the distal part of the femoral stem from the surrounding bone. We needed at least 2cm to have enough space for two locking pliers. This was achieved with a longitudinal osteotomy through the anterior cortex extending distally for 10cm (Figure 2A). The transversal osteotomy was 2cm below the tip of the femoral stump to allow enough space for two locking pliers. The transversal osteotomy was about 60 to 70 percent of the total bone circumference as shown (Figure 2B). A lamina spreader was then inserted in the osteotomy line proximally and two bone flakes were opened like a book in order to provide space for the tip of the locking pliers (Figure 2B). The proximal tip of the broken stem was compressed as much as possible with two locking pliers. A lamina spreader was then inserted in the remaining distal longitudinal osteotomy and carefully opened for 1 to 2mm to allow the bone to separate from the stem, avoiding fracture of the femur (Figure 2C). Simultaneously, a hammer was used on the locking pliers and with several strong strokes the broken stem was removed from the bone easily (Figure 3). Next, a cerclage wire was inserted below the horizontal cut to prevent a possible uncontrolled fracture of the femoral bone during broaching. A second cerclage wire was inserted on the proximal 2cm of the femoral bone, which adapted perfectly. The femoral canal was reconstructed perfectly with minimal damage to the periosteum. The new revision prosthesis was then inserted in the standard manner (Figure 4).

## Discussion

Due to the lifespan extension and better survival of patients with malignant diseases treated with joint replacement, the need for revision surgery will continue to increase. One of the reasons for revision surgery is mechanical failure of the prosthesis. Although not a common occurrence, the fractures of femoral stems have been previously described [2-6]. Factors predisposing to this form of stem failure include excessive patient weight, high levels of physical activity, deficient osseous support, malposition or loosening of the stem, the presence of a stress riser, and a reduced cross-sectional area within the stem [5,7-10]. Sotereanos *et al.* found two fractures of the stem in 122 patients (1.6 percent) using an extensively coated single-sized cobalt-chrome femoral component [3]. Lakstein *et al.,* in a study of 72 hips at five to 10 years of follow-up, reported one stem fracture at the modular junction [6]. Paprosky *et al.* found 6 percent of femoral component fractures after revision hip arthroplasty [2]. Efe *et al.* reported mechanical failure of four noncemented modular revision stems over a period of 28 months [11]. Various techniques have been developed for the removal of a well-fixed cementless femoral stem [1,12-15]. Glassman *et al.* used a technique with trephine reamers for stable implants and required interface access and division before their removal. Minimal bone damage was incurred, and in no case was reconstruction precluded by stem removal. There were no unplanned cortical perforations but two minor femoral fractures occurred [1]. Babis *et al.* also used a technique described by Glassman with motorized trephine reamers in two cases of fracture of femoral stem. They experienced one wear of the cutting heads, and one perforation to the posterior cortex of the femur [15]. Kim *et al.* used a microsagittal saw after creating a cortical window and this method can be applied to any kind of cementless stem and has the advantage of preserving the proximal portion of bone but necessitates special microsaws and the creation of a cortical window [12]. Moreland *et al.* also described the window technique for the removal of a fractured femoral stem [14]. Tanaka *et al.* used trephine reamers in removing a fractured femoral component in knee megaprostheses following tumor resection [13]. Although they are useful, they all have limitations and disadvantages. These techniques for the removal of a broken femoral stem complicated by femoral perforation or fracture have required the creation of a cortical window or have not been sparing of the remaining bone stock. The technique with trephine reamers is time-consuming and bone must be irrigated to avoid heat damage [15]. They are also complicated by using surgical instrumentary that is not standard and available at any operating room, such as a microsagittal saw [12] or special hollow trephines [1,13,15]. The precise and useful technique of revision surgery in the case of a fractured prosthesis is very important in order to conserve bone stock, thus achieving good revision results, preserving hip function and avoiding complications. Our technique is a simple and effective way of contesting the two main technical issues in dealing with a fractured prosthesis. First, it provides a facile and strong grip of the stem with wide-grip pliers, and second, it enables the hammering out of the stem with little resistance. It is time sparing and conserves enough bone stock, which is scarce enough in any revision surgery, especially after large tumor resections. Our technique can be easily performed with ordinary surgical instrumentary; technically, it is simple and reproductive. It can be applied to fractured femoral stems as well as for intramedullary nails regardless of the cross section of the implant. Postoperative bone healing is promoted by the excellent adaptation of the osteotomy line. Damage to the periosteum is only done during osteotomy through the anterior cortex and is minimized, which also promotes bone healing. Good fixation of the new revision prostheses can be made due to bone stock conservation and the excellent adaptation of the osteotomy line. This enables early mobilization and rehabilitation of the patient, minimizing the postoperative complications.

## Conclusion

We developed a simple and easy technique which we will continue to use because it can be applied to all fractured femoral stems and intramedullary nails regardless of their cross section, and to fractured femoral stems that do not stick out from the remaining bone. This technique can be performed with ordinary instruments, and it is sparing of the remaining bone stock.

## Consent

Written informed consent was obtained from the patient for publication of this case report and any accompanying images. A copy of the written consent is available for review by the Editor-in-Chief of this journal.

## Competing interests

The authors declare that they have no competing interests.

## Authors’ contributions

All authors participated in the development of the technique. All authors were involved in treating and operating on the patient. All authors participated in preparing the manuscript and all authors read and approved the final manuscript.
